# Presurgical temporal lobe epilepsy connectome fingerprint for seizure outcome prediction

**DOI:** 10.1093/braincomms/fcac128

**Published:** 2022-05-17

**Authors:** Victoria L Morgan, Lucas E Sainburg, Graham W Johnson, Andrew Janson, Kaela K Levine, Baxter P Rogers, Catie Chang, Dario J Englot

**Affiliations:** Institute of Imaging Science, Department of Radiology and Radiological Sciences, Vanderbilt University Medical Center, 1161 21st Avenue South, R0102 MCN, Nashville, TN 37232, USA; Department of Biomedical Engineering, Vanderbilt University, Nashville, TN 37235, USA; Department of Neurological Surgery, Vanderbilt University Medical Center, Nashville, TN 37212, USA; Institute of Imaging Science, Department of Radiology and Radiological Sciences, Vanderbilt University Medical Center, 1161 21st Avenue South, R0102 MCN, Nashville, TN 37232, USA; Department of Biomedical Engineering, Vanderbilt University, Nashville, TN 37235, USA; Institute of Imaging Science, Department of Radiology and Radiological Sciences, Vanderbilt University Medical Center, 1161 21st Avenue South, R0102 MCN, Nashville, TN 37232, USA; Department of Biomedical Engineering, Vanderbilt University, Nashville, TN 37235, USA; Department of Neurological Surgery, Vanderbilt University Medical Center, Nashville, TN 37212, USA; Institute of Imaging Science, Department of Radiology and Radiological Sciences, Vanderbilt University Medical Center, 1161 21st Avenue South, R0102 MCN, Nashville, TN 37232, USA; Institute of Imaging Science, Department of Radiology and Radiological Sciences, Vanderbilt University Medical Center, 1161 21st Avenue South, R0102 MCN, Nashville, TN 37232, USA; Institute of Imaging Science, Department of Radiology and Radiological Sciences, Vanderbilt University Medical Center, 1161 21st Avenue South, R0102 MCN, Nashville, TN 37232, USA; Department of Biomedical Engineering, Vanderbilt University, Nashville, TN 37235, USA; Institute of Imaging Science, Department of Radiology and Radiological Sciences, Vanderbilt University Medical Center, 1161 21st Avenue South, R0102 MCN, Nashville, TN 37232, USA; Department of Biomedical Engineering, Vanderbilt University, Nashville, TN 37235, USA; Department of Electrical Engineering and Computer Science, Vanderbilt University, Nashville, TN 37235, USA; Institute of Imaging Science, Department of Radiology and Radiological Sciences, Vanderbilt University Medical Center, 1161 21st Avenue South, R0102 MCN, Nashville, TN 37232, USA; Department of Biomedical Engineering, Vanderbilt University, Nashville, TN 37235, USA; Department of Neurological Surgery, Vanderbilt University Medical Center, Nashville, TN 37212, USA; Department of Electrical Engineering and Computer Science, Vanderbilt University, Nashville, TN 37235, USA

**Keywords:** seizures, surgery, magnetic resonance imaging, network, outcome

## Abstract

Temporal lobe epilepsy presents a unique situation where confident clinical localization of the seizure focus does not always result in a seizure-free or favourable outcome after mesial temporal surgery.

In this work, magnetic resonance imaging derived functional and structural whole-brain connectivity was used to compute a network fingerprint that captures the connectivity profile characteristics that are common across a group of nine of these patients with seizure-free outcome. The connectivity profile was then computed for 38 left-out patients with the hypothesis that similarity to the fingerprint indicates seizure-free surgical outcome. Patient profile distance to the fingerprint was compared with 1-year seizure outcome and standard clinical parameters. Distance to the fingerprint was higher for patients with Engel III–IV 1-year outcome compared with those with Engel Ia, Ib-d, and II outcome (Kruskal–Wallis, *P* < 0.01; Wilcoxon rank-sum *p*_corr_ <0.05 Bonferroni-corrected). Receiver operator characteristic analysis revealed 100% sensitivity and 90% specificity in identifying patients with Engel III–IV outcome based on distance to the fingerprint in the left-out patients. Furthermore, distance to the fingerprint was not related to any individual clinical parameter including age at scan, duration of disease, total seizure frequency, presence of mesial temporal sclerosis, lateralizing ictal, interictal scalp electroencephalography, invasive stereo-encephalography, or positron emission tomography. And two published algorithms utilizing multiple clinical measures for predicting seizure outcome were not related to distance to the fingerprint, nor predictive of seizure outcome in this cohort. The functional and structural connectome fingerprint provides quantitative, clinically interpretable and significant information not captured by standard clinical assessments alone or in combinations. This automated and simple method may improve patient-specific prediction of seizure outcome in patients with a clinically identified focus in the mesial temporal lobe.

## Teaser

Mesial temporal lobe epilepsy presents a unique situation where confident localization of the seizure focus may not result in a favourable outcome after surgery. Morgan *et al*. demonstrate an MRI connectome fingerprint that represents a network pattern across the brain that is associated with favourable seizure outcome in these patients.

## Graphical Abstract

**Graphical Abstract:**
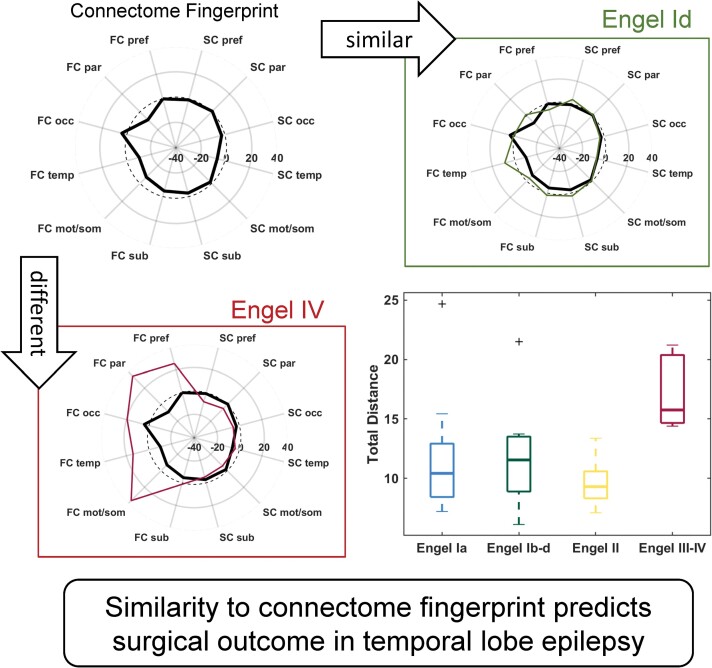

See Keller (https://doi.org/10.1093/braincomms/fcac158) for a scientific commentary on this article.

## Introduction

Mesial temporal lobe epilepsy (TLE) is the most common form of focal epilepsy. Its diagnosis is based on the identification of a seizure focus in the mesial temporal lobe using standard clinical measures^1^ and is associated with one of the highest seizure-free rates after resection at around two-thirds of patients.^2^ However, even as technology has improved our ability to localize the seizure focus in these patients, the rates of seizure freedom after surgical resection of the focus have not improved proportionally over the last several decades.^3,4^ This suggests that clinical assessments aimed at identifying the focus are not enough to fully characterize these patients and distinguish the fraction of patients that will not benefit from surgical intervention of the mesial temporal lobe.

But focal epilepsy is now considered a network disorder,^5,6^ with detectable effects across much of the brain well beyond the focus.^7^ MRI connectomics is a method to quantify networks using functional and structural MRI. Functional MRI uses synchronization of low frequency spontaneous blood oxygenation levels as indicators of functionally connected regions.^8^ Diffusion MRI exploits the anisotropic diffusion of water along white matter to track structural connections between regions.^9^ In fact, there is much evidence for widespread network alterations in patients with TLE measured using both functional^10,11^ and structural^12,13^ MRI connectomics.

Taken further, these network alterations have been successfully associated with patient-specific seizure outcome. As this literature grows, some general approaches to this challenge are emerging. Many studies have taken the approach comparing connectomes directly between seizure-free patients and those in whom seizures recur.^14–17^ While promising, these do not account for the belief that seizure recurrence can have diverse mechanisms^18,19^ that may lead to heterogeneous reasons for surgical failure. This may reduce power and lead to findings dependent on the particular pathophysiology represented in this seizure recur group, and less generalizable to a different cohort. Others compute a quantification of disease burden by identifying abnormalities throughout the connectome based on the assumption that number,^20^ and/or location and magnitude^21–23^ of network abnormalities is most associated with seizure recurrence. This approach allows for heterogeneity within the seizure recur group, as well as the seizure-free group, which may improve generalization to other patients with TLE, or possibly any focal epilepsy patient. However, it is not clear that every abnormality is related to seizure recurrence.

These approaches do not leverage the unique characteristics of the TLE patient group. In this work, we assume that the seizure-free group is homogeneous in such a way that all the patients respond favourably to similar surgical interventions targeting the mesial temporal lobe. We also assume that the seizure recur group is heterogeneous and does not possess the characteristics that make the seizure-free group homogeneous. To address this situation, we can utilize the idea of MRI connectome fingerprinting.^24,25^ Fingerprinting is used to describe a method where a single individual’s connectome will be more similar to their own connectome, even under different conditions (rest or tasks), than those from other individuals. We hypothesize that there exists a whole brain functional and structural connectome (SC) fingerprint of true unilateral, mesial TLE, such that similarity to the fingerprint will identify patients who will benefit from mesial temporal surgical intervention. We first proposed the idea of a ‘model’ network in an attempt to predict seizure outcome in a previous work.^26^ The results provided proof of concept in a small cohort of patients with TLE (*n* = 22) that functional and structural connectivity across a network of eight regions were more similar in those patients with favourable seizure outcome (Engel I–II),^27^ than unfavourable outcome (Engel III–IV).

In the present study, we built on the previous work with a larger, more diverse cohort of patients with TLE. Here we computed a whole-brain MRI connectome fingerprint of unilateral TLE using both functional and structural MRI. The accuracy of the fingerprint was assessed based on its ability to distinguish patients with different outcomes in a cohort of clinically similar patients. In addition, the comparisons of individual effects of connectivity type (functional versus structural) and measures of similarity (magnitude versus pattern) were investigated. To understand whether the fingerprint detected unique characteristics, similarity was compared with other clinical parameters and two existing outcome predictors. The objectives of these investigations were to further characterize the TLE connectome fingerprint, and to support its ability to provide information complementary to standard clinical assessments. If successful, this may provide an effective, interpretable addition to the presurgical assessment to improve patient-specific prediction of post-surgical seizure freedom by incorporating network information.

## Materials and methods

### Subjects

There were 52 patients with clinically diagnosed TLE [26 female, age (years): mean ± stdev = 39.88 ± 12.11, range 18–68] included in this study. They will be referred to as patients with TLE in this work. All were clinically diagnosed as unilateral mesial TLE based on presurgical evaluation including MRI, long-term video scalp EEG and interictal PET. Some patients also had invasive stereo-encephalography (SEEG). Exclusion criteria included structural abnormalities outside the mesial temporal lobe. All patients had either selective amygdalo-hippocampectomy, temporal lobectomy or mesial temporal laser ablation with at least 1-year seizure outcome assessment based on Engel outcome score. In addition, 85 healthy control subjects with no history of head injury or neuropsychiatric disease were included [41 female, age (years): mean ± stdev = 37.77 ± 13.58, range 18–71]. The protocol was approved by the Vanderbilt University Institutional Review Board. All participants gave informed consent.

To construct the TLE connectome fingerprint, we divided the 52 patients with TLE into three groups. The *model* group was made up of nine patients with Engel Ia seizure outcome up to and including last follow-up after surgery for at least 3 years. The purpose of this group was to identify the connectome characteristics that are consistent among patients with seizure-free outcome. The group purposely included heterogeneity of side of epilepsy and surgery type. The *testing* group consisted of five patients. Four of these had Engel Ia 1-year outcome, with three of these having Engel Ia seizure outcome up to and including last follow-up after surgery at least 2 years. The fourth had only 1 year since surgery. The fifth test patient had an Engel III 1-year outcome. The testing group also included a mix of side and type of surgery. These two groups were not intended to interrogate the entire parameter space. Instead, the purpose was to create a fingerprint based on *a priori* hypotheses using the model group and allow for initial tests of connectome parameters such as nodes of interest and weighting factors using the test group, to be described below. The remaining 38 patients made up the *left-out* group to evaluate the fingerprint in a completely independent data set. See Table 1 for clinical characteristics of each group.

**Table 1:** Unilateral TLE patient information by group and 1-year seizure outcome

Group	Model	Test	Left-out				
1-year outcome	Engel Ia	mixed	Engel Ia	Engel Ib-d	Engel II	Engel III–IV	*P*
# Patients	**9**	**5**	**15**	**11**	**5**	**7**	* *
Age (years: mean ± std)	42.3 ± 13.1	34.2 ± 18.8	45.4 ± 13.0	34.1 ± 9.8	38.0 ± 5.4	39.2 ± 5.0	0.10^1^
Sex (#F, %)	7, 77.7%	3, 60%	4, 26.6%	7, 63.6%	3, 60%	2, 28.5%	0.19^2^
Handedness (# Right, %)	8, 88.8%	3, 60%	11, 73.3%	11, 100%	4, 80%	6, 85.7%	0.35^2^
Side (# Right, %)	6, 66.6%	3, 60%	10, 66.6%	9, 81.8%	5, 100%	3, 42.8%	0.12^2^
Age of onset (years: mean ± std)	16.0 ± 14.9	16.5 ± 26.7	24.3 ± 16.8	13.4 ± 9.1	23.2 ± 12.3	22.7 ± 12.6	0.21^1^
Duration (years: mean ± std)	26.3 ± 14.2	17.8 ± 15.4	21.0 ± 17.2	20.7 ± 13.6	20.4 ± 16.1	16.4 ± 14.3	0.93^1^
FBTCS (# yes, %)	4, 44.4%	3, 60%	7, 46.6%	7, 63.6%	3, 60%	4, 57.1%	0.87^2^
Seizure frequency (per month ± std)	6.3 ± 7.7	17.9 ± 29.2	10.9 ± 9.3	42.5 ± 70.8	17.2 ± 26.7	8.0 ± 10.2	0.21^1^
MRI MTS (# yes, %)	6, 66.6%	3, 60%	10, 66.6%	5, 45.4%	3, 60%	4, 57.1%	0.85^2^
PET lateralizing (# yes, %)	7, 77.7%	5, 100%	13, 86.6%	7, 63.6%	3, 60%	5, 71.4%	0.56^2^
Ictal EEG lateralizing (# yes, %)	9, 100%	5, 100%	13, 86.6%	9, 81.8%	4, 80%	5, 71.4%	0.93^2^
Interictal EEG lateralizing (# yes, %)	6, 66.6%	4, 80%	11, 73.3%	9, 81.8%	4, 80%	4, 57.1%	0.68^2^
Invasive recording (SEEG) (# yes, %)	0, 0%	0, 0%	3, 20%	4, 36.3%	0, 0%	1, 14.2%	0.41^2^
Type of surgery (selAH, TL, laser)	5, 4, 0	4, 1, 0	13, 1, 1	10, 1, 0	3, 2, 0	3, 3, 1	0.15^2^

std = standard deviation, Side = side of epileptogenic zone, F = females, # = number, % = percent of patients in the group (model, test, left-out), R = right, FBTCS = focal to bilateral tonic–clonic seizures, MTS = mesial temporal sclerosis on MRI, SEEG = stereo-electroencephalography, selAH = selective amygdalo-hippocampectomy, TL = temporal lobectomy, laser = laser ablation, ^1^ = Kruskal–Wallis test between outcomes of the left-out group, ^2^ = Chi-square test between outcomes of the left-out group, *P* is *P*-value of statistical test between four outcome groups of the left-out data set.

### Imaging

All subjects underwent the same imaging procedure on a 3 Telsa MRI scanner with a 32-channel head coil (Philips Healthcare, Best, Netherlands). As in our previous work,^28^ the image acquisition included the following: (i) T_1_-weighted MRI for inter-subject normalization and regional and tissue segmentation (1 × 1 × 1 mm), (ii) T_1_-weighted MRI acquired in the same slice orientation as the functional images (1 × 1 × 3.5 mm with 0.5 mm gap), (iii) functional T_2_*-weighted MRI at rest with eyes closed (34 axial slices, echo time = 35 ms, repetition time = 2 sec, 3 × 3 × 3.5 mm with a 0.5 mm gap, 10 min), (iv) diffusion MRI for structural connectivity (50 slices, 2.5 × 2.5 × 2.5 mm, 92 directions, b = 0, 1600 s/mm^2^). Cardiac and respiratory fluctuations were recorded at 500 Hz using the scanner integrated pulse oximeter and respiratory belt. Scan session was within 6 months of the surgery except in four patients whose surgeries were between 6 and 16 months of scan.

### Connectome development

The T_1_-weighted images were used to segment the brain into 117 regions of interest (nodes) using two atlases. First, 56 cortical and subcortical nodes in each hemisphere and the brainstem were identified using an in-house developed Multi-Atlas algorithm.^29,30^ Then, the subfields of the left and right hippocampus were identified using FreeSurfer 6 suite.^31^ These subfields were used to form composite anterior and posterior hippocampal nodes according to McHugo *et al.*^32,33^

Preprocessing and connectome development followed procedures published previously^28^ and reiterated here. The functional MRI images were preprocessed using SPM12 software (http://www.fil.ion.ucl.ac.uk/spm/software/spm12/) and MATLAB 2019a (The MathWorks, Inc, Natick, MA). First, physiological noise correction using the pulse oximeter and respiratory belt data was performed using the Retrospective Correction of Physiological Motion Effects in functional MRI (RETROICOR) protocol.^34^ Next, slice timing correction, motion correction, spatial normalization to the Montreal Neurological Institute template via the T_1_-weighted data sets, and spatial smoothing (6 × 6 × 6 mm FWHM Gaussian kernel) was implemented in SPM12. Finally, the functional MRI time series were temporally band-pass filtered at 0.0067 to 0.1 Hz.^35^

The preprocessed functional MRI time series were averaged across all voxels in each node to create nodal time series. Then the partial Pearson correlation was computed between each pair of nodes using six motion and one mean white matter time series as confounds, and then normalized using the Fisher Z transform.^36^ This resulted in a 117 × 117 matrix of functional connectivity values for each subject. These connections between nodes are referred to as edges. Then, to correct for the effects of age, the linear fit and the root mean squared error of the fit to age across all the control subjects were computed for each edge. Then the functional connectivity for each patient was corrected for age through linear regression and divided by root mean squared error of the linear fit of each edge. This resulted in a 117 × 117 functional connectome (FC) for each patient in units of standard deviations from age-matched control. This connectome was originally computed in native left and right hemispheres, but patients were then transformed into ipsilateral and contralateral with respect to seizure focus.

The diffusion MRI images were preprocessed using PreQual,^37^ an automated pipeline, including denoising,^38^ eddy current and motion correction,^39^ and bias correction of B1 field inhomogeneity.^40^ Then the response function was estimated for spherical deconvolution for estimation of fibre orientation distribution.^41^ After preprocessing, SPM12 and MATLAB 2019a were used to generate the grey matter–white matter interface using the T_1_-weighted image and the mean B0 image. Using MRtrix3,^40^ 2 × 10^7^ anatomically constrained probabilistic streamlines were generated through the white matter from this interface.^42^ The streamlines were then reduced to 1 × 10^7^ using spherical convolution informed filtering to match the fibre orientation density integrals (SIFT2).^43^ The 117 nodes were then used to create a matrix of the streamline count between each pair of nodes scaled by the inverse of the two node volumes as a measure of structural connectivity between nodes. In addition, similar to functional connectivity, the structural connectivity measures were corrected for age using linear fits of the healthy control data. However, to convert these data to a Gaussian distribution a log transform was used to prior to the fitting. This resulted in 117 × 117 SCs in units of standard deviation from age-matched control for each patient. These were computed in left and right hemispheres and were then converted to ipsilateral and contralateral to seizure focus.

### Fingerprint development

The connectomes of the nine model patients were used to compute a larger sample that represented their distribution. This was computed via 10 bootstrap samples of six patients each. From each sample, four subsample connectomes were created using the mean +/− up to four times the standard deviation of each edge of the six patients in the sample. This process resulted in 40 FCs and SCs to be used as the model connectomes to create the fingerprint.

We first reduced the FC and SC information to summary parameters by computing the weighted degree of each node as its sum to all nodes designated as nodes of interest (to be explained later) multiplied by a weight. Then the weighted degree was averaged over major subregions (‘lobes’) of the brain: prefrontal, parietal, occipital, temporal, sensory/motor, and subcortical. To further avoid overfitting and reduce the number of parameters, only the six ipsilateral lobes were included. Thus, for training 40 functional and 40 structural vectors of length six were computed. These functional and structural connectivity vectors are a representation of a connectivity profile of an individual and can be visualized on a polar plot.

The TLE fingerprint, a specific connectivity profile that represents seizure-free outcome, was developed to which other individual patients could be compared using quantification of similarity measures. The goal was to use the model and test groups find a set of fingerprint parameters in which (i) the four Engel Ia subjects of the test data were more similar (less total distance) to the fingerprint than the maximum distance of all the model subjects (no false outliers), and (ii) the test subject with Engel III outcome had greater distance than all model and other test subjects (one true outlier). With these, we evaluated two parameter choices. First, we evaluated the nodes of interest used in the weighted degree computation. For this we evaluated using all 117 nodes versus using only 14 *a priori* nodes representing the regions used in our previous work.^26^ The 14 nodes include ipsilateral and contralateral middle cingulate gyrus, precuneus, thalamus, anterior insula, posterior insula, anterior hippocampus and posterior hippocampus. Second, we evaluated equal versus unequal weighting for ipsilateral and contralateral nodes in the weighted degree computation.

To quantify similarity from the fingerprint, and assess our model and test subjects, we used two distance measures. First, Euclidean distance to the mean of the computed 40 model profiles represented the magnitude of the difference between the individual patient and the fingerprint. Second, the Mahalanobis distance^44^ from the set of 40 model profiles quantified a measure of pattern similarity by also taking into account the covariance between the values of the six lobes of the training data. Functional distance was then computed as Euclidean + Mahalanobis distance from the FC fingerprint profile. The same was done for structural distance. These can be plotted on the x and y axes to visualize similarity due to each type of connectivity. Total distance was computed from the origin of this plot as the square root of the sum of the squares of functional and structural distance.

### Statistical analysis

#### Seizure outcome

The similarity to the final TLE fingerprint was then compared with seizure outcome in the left-out group of patients. The patients were divided into four groups based on Engel outcome at 1 year post-surgery—Engel Ia, Engel Ib-d, Engel II, Engel III–IV. A Kruskal–Wallis test was used to determine differences in total distance between the groups. Individual groups were then compared using the Wilcoxon rank-sum test corrected for the six tests between all pairs using Bonferroni correction. The same tests were also used to compare Mahalanobis distance alone, Euclidean distance alone, functional distance alone and structural distance alone to determine the contributions of these different measures. Finally, the receiver operating characteristic curve^45^ was computed using total distance to predict Engel III–IV outcome. From this curve we report sensitivity, specificity and area under the curve.

Since the 14 nodes of interest were chosen *a priori* as explained in our previous work,^26^ we performed a *post hoc* analysis to examine whether other combinations of 14 nodes might better distinguish patients with Engel III–IV seizure outcome in the left-out group. To do this, we performed 5000 iterations each sampling 14 random nodes from the total 117. In each iteration, a random fingerprint was computed and the distance for each of the left-out subjects was determined. The subjects were then ranked based on total distance with Rank 1 having the lowest total distance to the given randomly created fingerprint. Then the rankings for the subjects with an unfavourable (Engel III–IV) outcome were summed. This gives a single value per iteration where higher values indicate the rankings of the patient with Engel III–IV outcome are higher for that set of 14 random nodes, which is desirable. This provided a null distribution to which the ranking of the real TLE fingerprint could be compared, and, therefore, a quantification of the validity of our hypothesized set of 14 nodes.

#### Clinical parameters and predictors

It is important to understand whether there are specific clinical parameters that influence the distance of the patient from the TLE fingerprint. In the left-out patients, the relationship between total distance to fingerprint and each of the following were compared using the Spearman correlation: age at time of scan (years), age of onset (years), duration of disease (years), frequency of focal aware seizures, (per month), frequency of focal impaired awareness seizures (per month), frequency of focal to bilateral tonic–clonic seizures (per month), and total seizure frequency (per month). The relationship of the distance to the fingerprint between patients with and without the following were compared using the Wilcoxon rank-sum test: lateralizing ictal EEG, lateralizing interictal EEG, invasive recording (SEEG), presence of mesial temporal sclerosis on MRI, and lateralizing PET. Statistics were corrected for multiple comparisons using Bonferroni correction.

However, seizure outcome prediction after epilepsy surgery may be best predicted using a multi-variate approach; and, indeed, there are a few published tools available to make these predictions. To assess the unique outcome prediction ability of the TLE fingerprint the distance to the fingerprint was compared to two published outcome prediction scores. It should be noted that of these tools were designed for use on all patients with focal epilepsy, not only TLE. The first, the modified seizure freedom score^46^ provides a score from 1 to 6 with higher score having higher prediction of seizure freedom. The second, the epilepsy surgery nomogram^47^ predicts the probability of seizure freedom at 2 years post-surgery from 0 (lowest) to 1 (highest). The Spearman correlation across the left-out group between the prediction score and the total distance to the fingerprint was computed. In addition, the scores were compared between the four outcome groups—Engel Ia, Engel Ib-d, Engel II, and Engel III–IV—using the Kruskal–Wallis test.

### Data availability

The data that support this study are available from the corresponding author, upon reasonable request. The algorithms developed here can be found at https://github.com/vmorgan-lab/TLE_fingerprint.

## Results

Table 1 provides the clinical information of the patients in each of the three groups. The patients of the left-out group are listed by 1-year seizure outcome. Statistics were performed between these four outcome groups with none having significant difference based on Kruskal–Wallis or χ^2^ test.

### Fingerprint development

**Figure 1:**
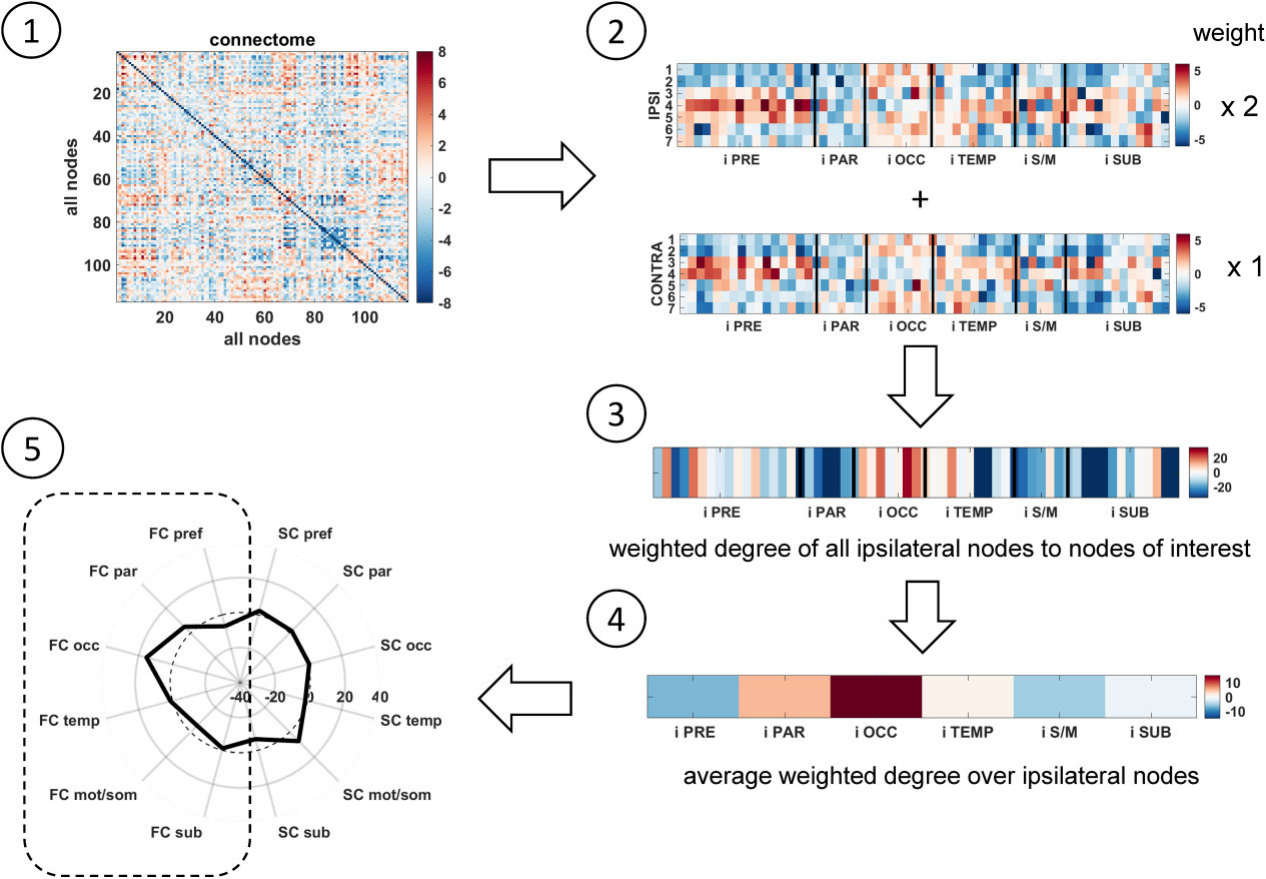
**Connectome to connectivity profile algorithm.** (**1**) Start with functional or structural connectome. (**2**) Consider only edges between ipsilateral nodes and the 14 nodes of interest. (**3**) Compute a weighted degree by weighting the seven ipsilateral nodes by two and seven contralateral nodes as one and summing across all ipsilateral nodes. (**4**) Average the ipsilateral weighted degree values across each of the six lobes of the brain. (**5**) Plot those on half of the polar plot to represent the connectivity profile. Repeat with the other connectome. The fingerprint is the profile that represents patients with seizure-free outcome. The same process is used to create the connectivity profile of each patient to be compared with the fingerprint. ipsi and i = ipsilateral to seizure focus; contra = contralateral to seizure focus; pref = prefrontal lobe; par = parietal lobe; occ = occipital lobe; temp = temporal lobe; mot/som = motor and sensory/motor lobe; sub = subcortical structures (all ipsilateral to seizure focus); FC = functional connectome distance; SC = structural connectome distance. Units are standard deviations from age-matched control.

The model and test groups were used to evaluate two parameters: (i) all 117 nodes as nodes of interest versus using 14 predetermined nodes based on previous work and (ii) weighting ipsilateral and contralateral nodes with 1 versus ipsilateral edges weight = 2 and contralateral nodes = 1. Each of these four scenarios were evaluated. Only the 14 nodes of interest and the 2 to 1 ipsilateral to contralateral weighting resulted in four test subjects with Engel Ia seizure outcome having lower distance than the maximum model distance, and the test subject with Engel III outcome having larger distance than all other training and test subjects. From these findings, the resulting steps of the creation of a connectivity profile for use as a fingerprint are illustrated in Fig. 1. Step 1 begins with a full connectome (FC or SC). Step 2 involves taking the subset of the connectome of all ipsilateral nodes to the 14 nodes of interest. In Step 3, a weighted degree is computed where the ipsilateral nodes (ipsilateral to ipsilateral edges) have weight = 2 and the contralateral nodes (contralateral to ipsilateral edges) have weight = 1. The weighted degree values are averaged over six ipsilateral lobes resulting in a vector of length six in Step 4. In Step 5, this vector can be visualized in half of a polar plot representing a connectivity profile. The process is repeated with the other connectome for the other six values in the other half of the profile. The same process is used to create the connectivity profile of each patient to be compared to the fingerprint. The units of the measures are standard deviations from age-matched control. This allows for more intuitive interpretation of each value.

**Figure 2:**
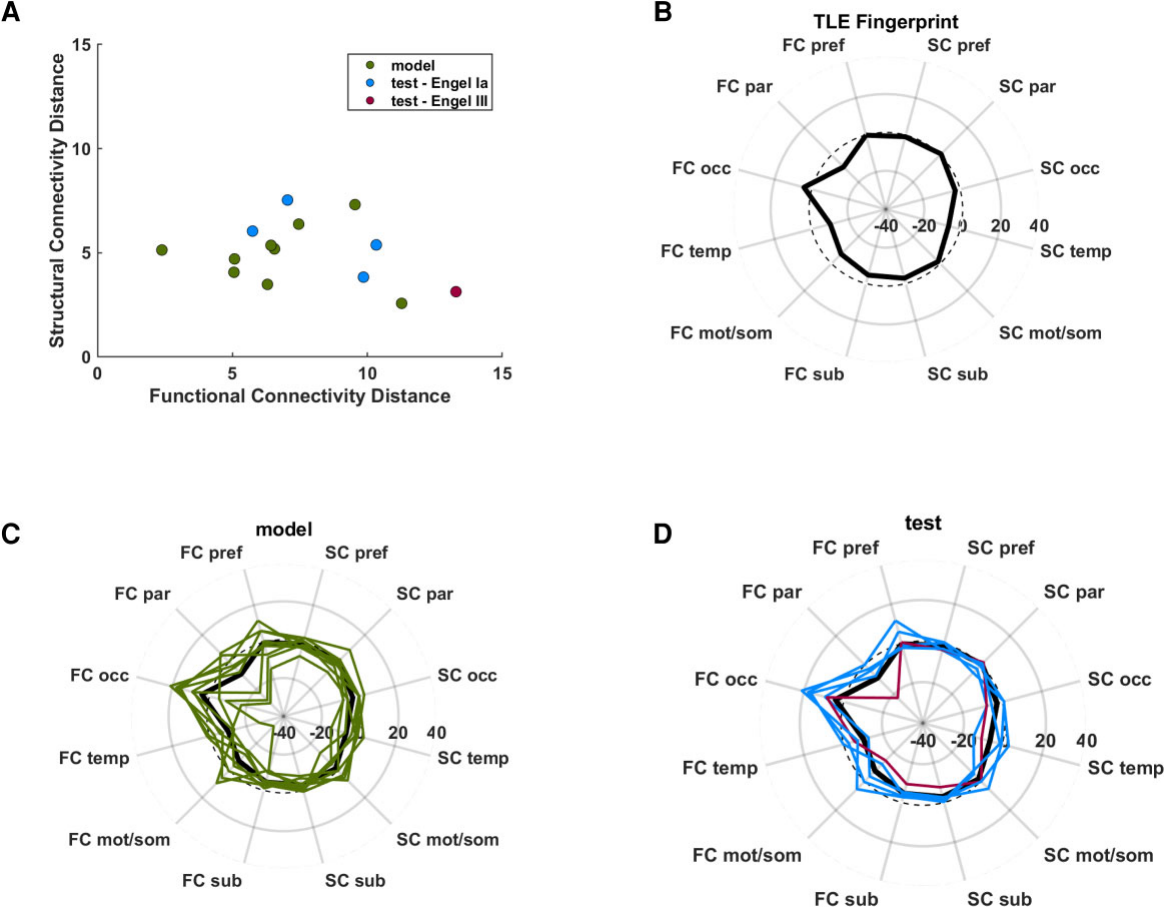
**TLE fingerprint development.** (**A**) Functional and structural distance plot of model and test data. (**B**) Polar plot of functional and structural connectivity profile of TLE fingerprint. (**C**) Connectivity profiles of nine model patients (green) and TLE fingerprint (black). (**D**) Connectivity profiles of five test patients (blue = Engel Ia, red = Engel III 1-year outcome) and TLE fingerprint (black). FC = functional connectome distance; SC = structural connectome distance; pref = prefrontal lobe; par = parietal lobe; occ = occipital lobe; temp = temporal lobe; mot/som = motor and sensory/motor lobe; sub = subcortical structures (all ipsilateral to seizure focus); dashed line = zero denoting age-matched control. Units are standard deviations from age-matched control. Values in A are distant from fingerprint. Values in B-D are connectivity measures from weighted degree.

Figure 2A shows the functional and structural distances of the model and test data with respect to the chosen TLE fingerprint. While the actual fingerprint from which the Mahalanobis distances are computed are a set of 40 permuted datasets taken from the model set, the average of those is shown in Fig. 2B. Each of the nine model subjects whose distances are shown in Fig. 2A are shown in Fig. 2C, with the test subjects shown in Fig. 2D. The test subject with the Engel III outcome is shown in red.

### Seizure outcome

**Figure 3:**
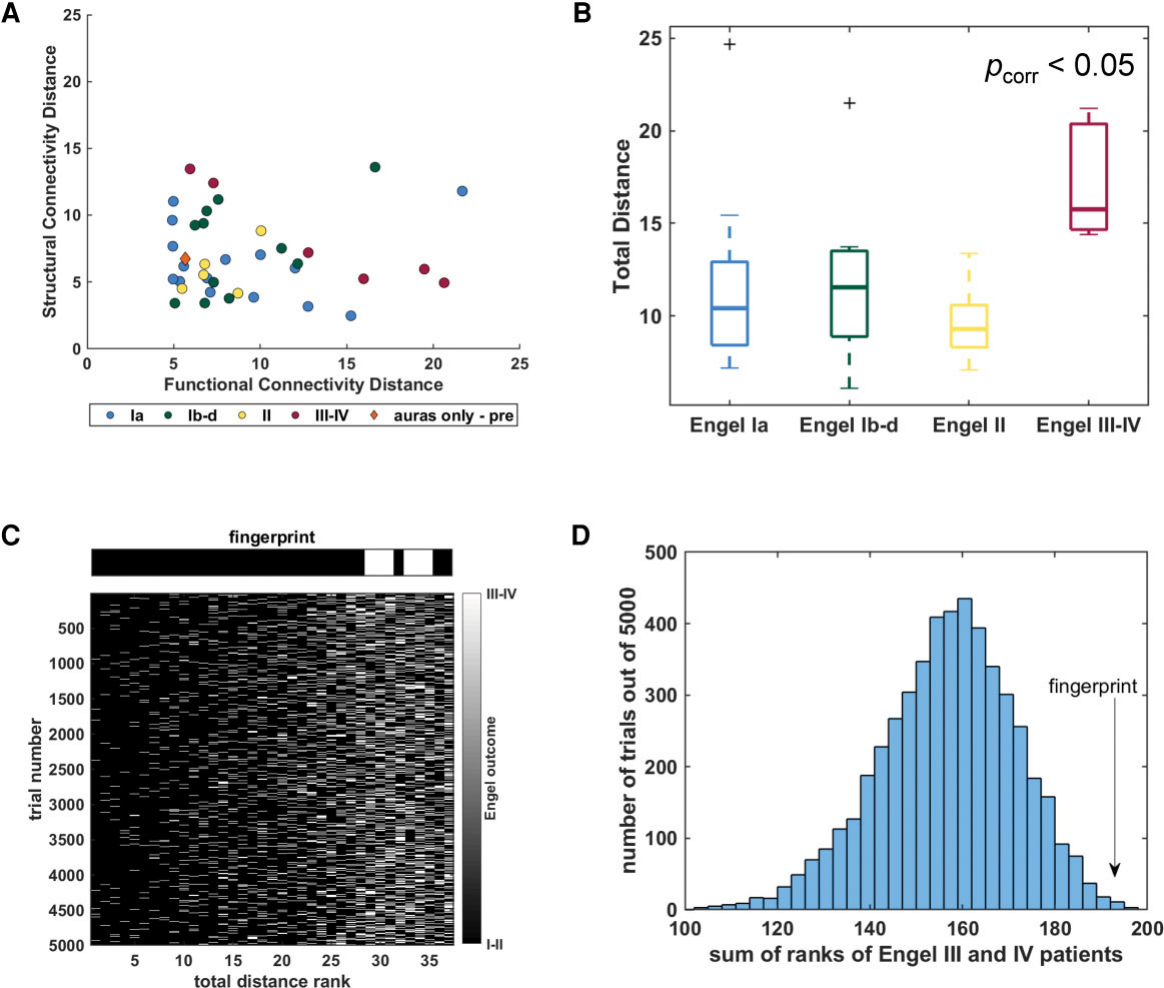
**TLE fingerprint related to 1-year seizure outcome in left-out group (*n* = 38).** (**A**) Functional and structural distance plot of left-out patients. Colour denotes 1-year Engel outcome. (**B**) Total distance across each outcome group. Patients with Engel III–IV 1-year outcome have greater distance than each of the other three outcome groups (Kruskal–Wallis *P* < 0.05, Wilcoxon rank-sum on individual pairs *p_corr_* < 0.05 Bonferroni correction for six tests). In A and B, units are standard deviations from age-matched control. (**C**) Rankings of patients with Engel III–IV outcome (white) and other patients (black) for each of the 5000 trials of randomized 14 nodes. Rankings using the TLE fingerprint shown above for comparison. (**D**) Histogram of sum of ranks of patients with Engel III–IV for each trial. The value of the TLE fingerprint sum (192) indicated by arrow with only five trials (0.1%) with greater values.

The functional and structural distance to the TLE fingerprint was computed for each of the patients in the left-out group (*n* = 38) (Fig. 3A). Note that there was one patient that was given an Engel III outcome (and is included as such in Table 1), who only experienced focal aware seizures (auras) before surgery and continued with no improvement after surgery. This patient is indicated with an orange diamond in this figure.

The total distance of each patient to the TLE fingerprint are compared in Fig. 3B, with the patient indicated by the orange diamond excluded (*n* = 37). These results show patients with Engel III–IV have greater distances than each of the other outcome groups (Kruskal–Wallis, *P* < 0.01; Wilcoxon rank-sum between individual groups, Engel III–IV greater than Engel Ia, Engel Ib-d and Engel II, *p*_corr_ <0.05 Bonferroni-corrected for six tests). When only functional or structural connectivity were utilized, there was no differences between the four outcome groups (Kruskal–Wallis, *P* > 0.05). When only Mahalanobis distance was used, the patients with Engel III–IV had greater distance than those with Engel Ia and with Engel II but not Engel Ib-d outcome (Kruskal–Wallis, *P* < 0.05; Wilcoxon rank-sum between individual groups, Engel III–IV greater than Engel Ia and Engel II, *p*_corr_ <0.05 Bonferroni-corrected for six tests). When using only Euclidean distance no individual pairs were significantly different with correction for multiple comparisons (Kruskal–Wallis, *P* < 0.05; Wilcoxon rank-sum between individual groups, *p*_corr_ >0.05 Bonferroni-corrected for six tests). Finally, the receiver operating characteristic curve analysis revealed that the TLE fingerprint had 100% sensitivity and 90% specificity (area under the curve = 0.9194) when using total distance to predict Engel III–IV outcome.

To compare the chosen 14 nodes of interest to a random sample of 14 nodes, we performed 5000 permutations using 1-year outcome. For each permutation, the subjects were ranked lowest distance to highest, and the rank of the patients with Engel III–IV outcome were summed. This assessment excluded the patient with presurgical auras only (*n* = 37). Fig. 3C shows the rankings for each trial in each row with white for patients with Engel III–IV outcome and black for the others. The rankings using the TLE fingerprint is shown above it. The histogram in Fig. 3D shows the ranking results of the 5000 trials. There were 5 random permutations with higher ranking than the TLE fingerprint (192), indicating that our *a priori* nodes outperformed 99.9% of the 5000 trials.

### Clinical parameters and predictors

When the correlation between total distance to the TLE fingerprint and individual clinical parameters was assessed in the left-out group, there were no significant correlations between age at scan (years), age of onset (years), duration of disease (years), frequency of focal aware seizures (per month), frequency of focal impaired awareness seizures (per month), frequency of focal to bilateral tonic–clonic seizures (per month), and total seizure frequency (per month) (Spearman correlation, *p*_corr_ >0.05). There was no difference in total distance to the fingerprint between patients with and without lateralizing ictal EEG, lateralizing interictal EEG, invasive recording (SEEG), presence of mesial temporal sclerosis on MRI and lateralizing PET (Wilcoxon rank-sum, *p*_corr_ >0.05).

**Figure 4:**
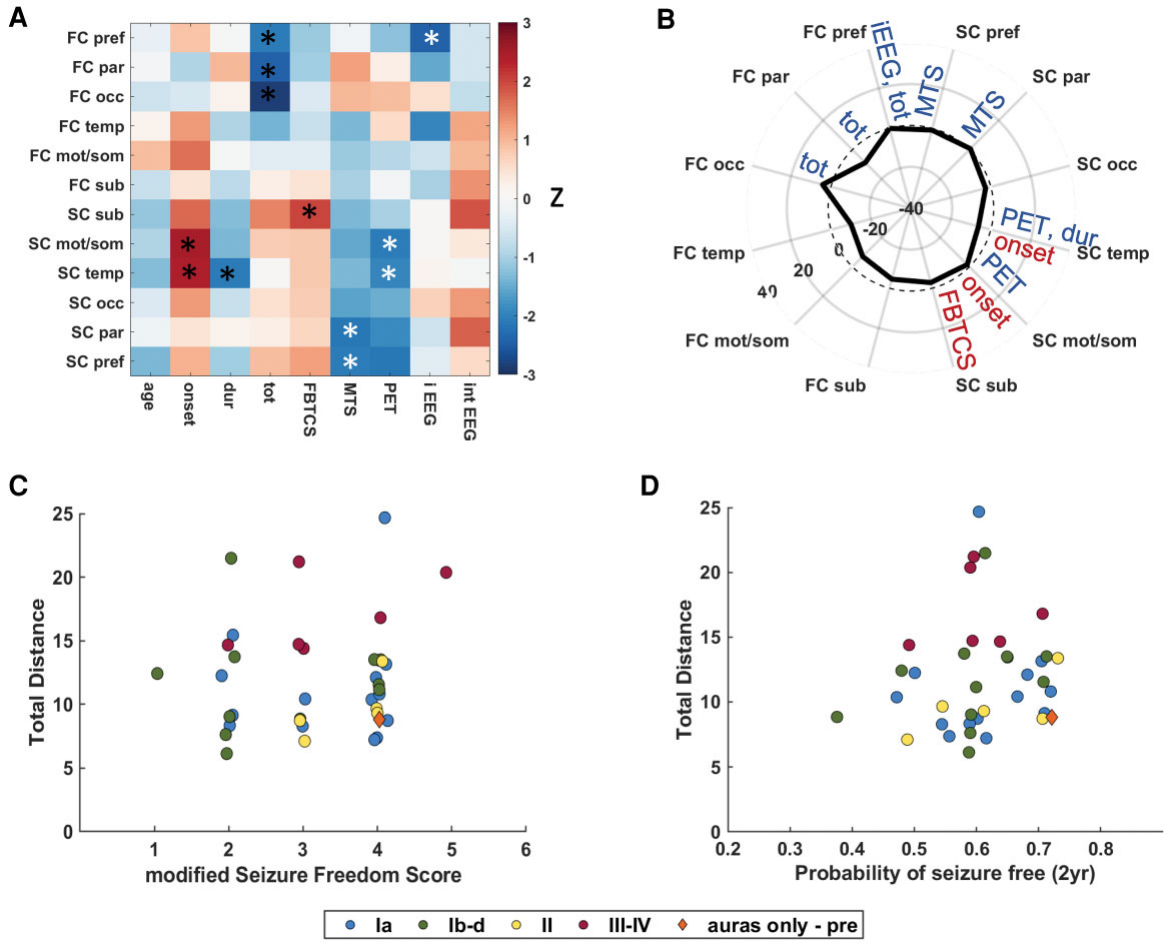
**TLE fingerprint related to clinical parameters and outcome predictors.** (**A**) Individual measures of the TLE fingerprint related to clinical parameters. Black and white asterisks indicate Spearman correlation, *p*_unc_ <0.05 and Wilcoxon rank-sum, *p*_unc_ < 0.05, respectively. (**B**) TLE fingerprint with relationships from A indicated along each connectivity parameter. Red font indicate positive clinical parameter change with increase in connectivity, and blue font indicates negative parameter change with increase in connectivity. For binary measures (i.e. EEG, PET, and MTS) blue font indicates decrease in connectivity with presence of parameter (i.e. lateralizing PET). FC = functional connectome distance; SC = structural connectome distance; pref = prefrontal lobe; par = parietal lobe; occ = occipital lobe; temp = temporal lobe; mot/som = motor and sensory/motor lobe; sub = subcortical structures (all ipsilateral to seizure focus); tot = total seizure frequency per month; onset = age of onset (years); dur = duration of disease (years); FBTCS = frequency of focal to bilateral tonic–clonic seizures per month; MTS = mesial temporal sclerosis on MRI; PET = lateralizing PET; iEEG = lateralizing ictal EEG; int EEG = lateralizing interictal EEG; dashed line = zero denoting age-matched control. Units are standard deviations from age-matched control. (**C**) Total distance to the TLE fingerprint is not linearly related to modified Seizure Freedom Score^46^ (Spearman correlation, ρ = 0.08, *P* > 0.05). (**D**) Total distance to the TLE fingerprint is not linearly related to the probability of 2-year seizure freedom nomogram score^47^ (Spearman correlation, ρ = 0.20, *P* > 0.05). Total distance is in standard deviations from age-matched control.

Since no relationships were detected between the total distance to the TLE fingerprint and clinical parameters, we also computed correlation between the same clinical parameters and each of the 12 measures in the TLE fingerprint separately—functional and structural ipsilateral prefrontal, parietal, occipital, temporal, sensory/motor, and subcortical region groups. In the 38 left-out patients, functional prefrontal, parietal and occipital connectivity to the nodes of interest decreases as number of total seizures increases (Spearman correlation, *p*_unc_ <0.05). The functional connectivity to the prefrontal lobe was decreased in patients where ictal EEG was lateralizing (Wilcoxon rank-sum, *p*_unc_ <0.05). The structural connectivity to the prefrontal and parietal lobes was decreased in patients with mesial temporal sclerosis on MRI (Wilcoxon rank-sum, *p*_unc_ <0.05). The structural connectivity to the temporal and sensory/motor regions was decreased in patients with lateralizing PET (Wilcoxon rank-sum, *p*_unc_ <0.05) and increased as age of onset increased (Spearman correlation, *p*_unc_ <0.05). The structural connectivity to the temporal region decreased as duration of disease increases (Spearman correlation, *p*_unc_ <0.05). The structural connectivity to the subcortical regions increased as the number of focal to bilateral tonic–clonic seizures increased (Spearman correlation, *p*_unc_ <0.05). (Figs. 4A and B)

Two seizure outcome prediction scores, the modified seizure freedom score^46^ and the probability of seizure freedom from the epilepsy nomogram,^47^ were not correlated with total distance to the fingerprint (Spearman correlation, *P* > 0.05). (Figs. 4C and D) Neither score was different between the four outcome groups at either 1 or 3 years after surgery (Kruskal–Wallis, *P* > 0.05).

## Discussion

**Figure 5:**
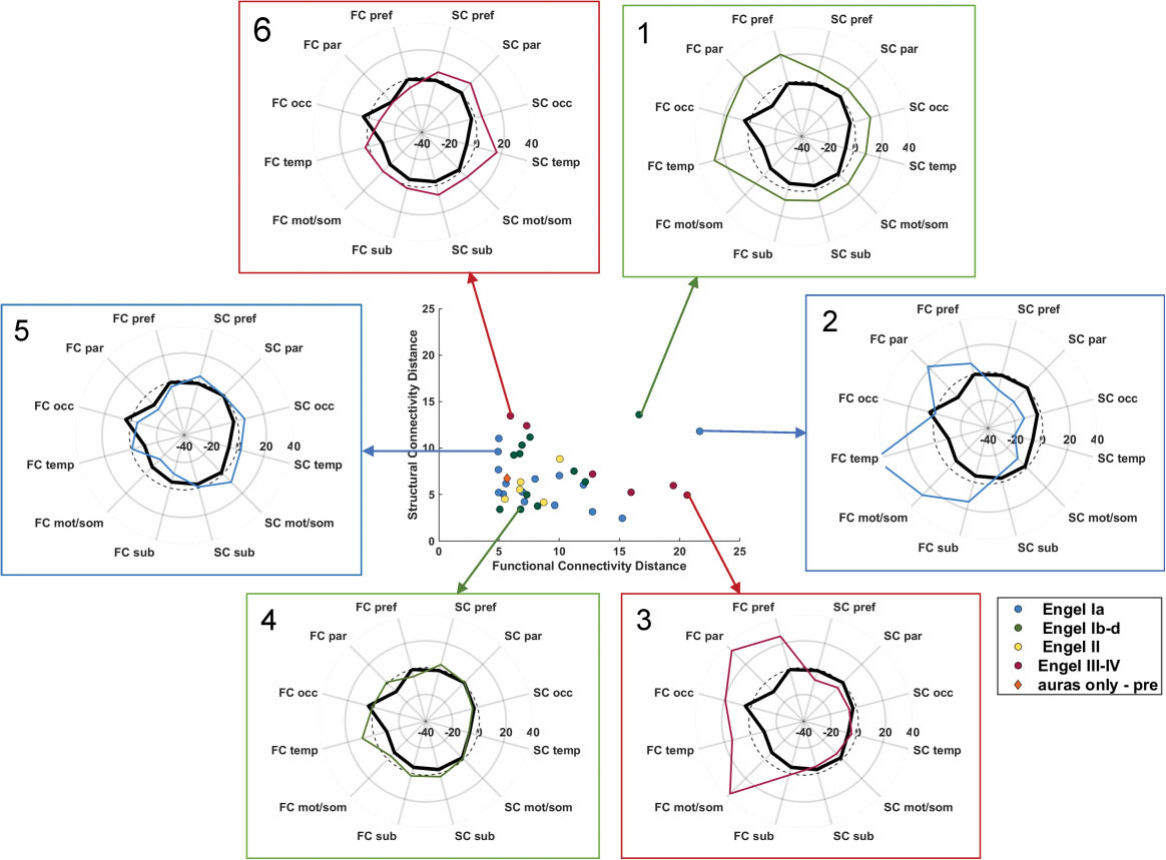
**Examples of patient connectivity profiles.** Profiles computed as described in Fig. 1. Centre plot is same as Fig. 3A. All outcomes are at 1 year after surgery. (**1**) Outlier patient with Engel Id outcome after right selAH, (**2**) outlier patient with Engel Ia outcome after left selAH, (**3**) patient with Engel IV outcome after left laser ablation, (**4**) patient with Engel Id outcome after right selAH, (**5**) patient with Engel Ia outcome after right temporal lobectomy, and (**6**) patient with Engel IIIa outcome after left temporal mesial and temporal pole resection. selAH = selective amygdalo-hippocampectomy. Units are standard deviations from age-matched control. Note other profiles of patients are shown in Figs. 2C and D for comparison. FC = functional connectome distance; SC = structural connectome distance; pref = prefrontal lobe; par = parietal lobe; occ = occipital lobe; temp = temporal lobe; mot/som = motor and sensory/motor lobe; sub = subcortical structures (all ipsilateral to seizure focus); dashed line = zero denoting age-matched control.

In this work, we present an automated, quantitative, and visually interpretable functional and structural connectivity fingerprint of TLE in which similarity to the fingerprint predicts Engel I–II seizure outcome 1 year after mesial temporal resection with 100% sensitivity and 90% specificity. The fingerprint was developed using nine patients with TLE with Engel Ia 1-year outcome and then tested on 38 patients in a totally independent data set. It is theorized that in practice the connectivity profile (examples in Figs. 2 and 5) of an individual patient being considered for mesial temporal surgery can be computed and compared to the TLE connectivity fingerprint. They can be visually evaluated for similarity and distances can be quantified and compared with other patients. If distances are higher than the other patients with Engel I–II outcome, more localization testing or other treatments may be considered.

### Seizure outcome

While patients with Engel III–IV 1-year outcomes had statistically increased distance to the fingerprint, it is noted that some had high functional connectivity distances, whereas others had high structural connectivity distances. In fact, when functional and structural connectivity distances were used individually, the pattern of increased distance in patients with Engel III–IV outcome was not detected. This is notable since in the test set, the patient with Engel III outcome had increased functional connectivity only. Similarly, when using only magnitude (Euclidean distance) or pattern (Mahalanobis distance) of similarity individually, the outcomes were not differentiated. This may reflect the heterogeneity in seizure recurrence in the first year after surgery.^18,19^

There are two outliers with Engel I–II 1-year outcome with high total distance (Figs. 3A and 5). First, there is a patient with Engel Id outcome after right selective amygdalo-hippocampectomy (Fig. 5, ‘1’). This patient had focal to bilateral tonic–clonic seizures only in the context of missed medication. The second patient (Fig. 5, ‘2’) that was classified as Engel Ia outcome underwent a 5-day long-term video EEG in the epilepsy monitoring unit 1 year after left selective amygdalo-hippocampectomy surgery. The results indicated 13 typical spells without EEG change, which were deemed non-epileptic. Further, there was evidence of non-specific left temporal cerebral dysfunction but no interictal epileptiform activity. It is unknown how these findings contributed to the change in that patient’s connectivity profile. In addition, we included a patient with only focal aware seizures (auras) before surgery (Fig. 2, orange diamond). While the designated outcome was considered only some improvement (Engel III), it is interesting to note that their distance to the fingerprint was similar to other patients whose seizures with loss of consciousness improved to only focal aware seizures.

With 6786 unique edges in each FC and SC, searching the entire parameter space for a best fit connectome would be subject to severe overfitting of the data and lack of generalization to other data sets. Therefore, we tested a hypothesis of 14 nodes of interest (seven in each hemisphere), known to be implicated in TLE and used in a previous proof of concept study^26^: anterior and posterior hippocampus, anterior and posterior insula, thalamus, precuneus, and mid cingulate gyrus. The fingerprint represents the connection of each of these regions connected to all other ipsilateral regions of the brain. In addition to evaluating on the left-out patients, we also compared with a null distribution of random sets of 14 nodes. That showed that the *a priori* nodes performed better than 99.9% of the random trials in the left-out data.

### Clinical parameters and predictors

Next, we evaluated if a particular clinical assessment was driving the distance measurement. There was no significant linear relationship between any of the parameters tested (listed in Table 1). This suggests that the fingerprint is not sensitive to any one clinical parameter and that there is no single clinical parameter that is related to seizure outcome in this patient cohort. But do TLE clinical parameters affect parts of the connectivity profile? We did detect weak relationships (significance uncorrected) between several measures within the connectivity profile and clinical parameters across the left-out group. Interestingly, functional connectivity from the nodes of interest to the ipsilateral frontal, parietal, and occipital lobes decreased as total seizure frequency increased. Evidence of mesial temporal sclerosis was associated with decreased structural connectivity to the prefrontal and parietal lobes, while lateralizing PET was associated with decreased structural connectivity to temporal and sensory/motor regions. Structural connectivity to the subcortical regions increased as number of focal to bilateral tonic–clonic seizures increased. These weak relationships imply that parts of the fingerprint are sensitive to characteristics of TLE, but the fingerprint as a whole is related to seizure outcome.

Additionally, the total distance to the model was not related to either the modified seizure freedom score^46^ nor the probability of seizure freedom nomogram score.^47^ Furthermore, the two prediction algorithms were applied to the left-out patients and resulted in inability to distinguish different outcome groups at 1 year after surgery. We interpret this as evidence that the two prediction algorithms may perform better in general population of patients with focal epilepsy, but in those patients where standard clinical assessments confidently identify the seizure focus in unilateral mesial temporal structures, connectivity may provide the next level of outcome prediction.

Based on these results, we propose an avenue of potential clinical utilization of this methodology, specifically in patients in which standard clinical presurgical assessments have diagnosed unilateral mesial TLE. We suggest that the network profile described here should be computed in the patient and compared to the fingerprint, both quantitatively, using the distance measures and qualitatively, by plotting the profile with the fingerprint (examples in Fig. 5). A similarity to the fingerprint suggests a favourable outcome and reduce the need for invasive testing. A large quantitative distance to the fingerprint may indicate a potential for less optimum outcome and prompt invasive testing and appropriate patient counselling. The regions of large distances in the qualitative profiles may indicate regions for further invasive or non-invasive testing, but this has not been studied. More validation will be needed, however, to bring this to the clinic.

### Considerations

It should be noted that (i) the fingerprint was developed using patients with Engel Ia 1-year outcome, and (ii) the similarity to the fingerprint was computed for patients grouped by four different outcomes—Engel Ia, Engel Ib-d, Engel II, and Engel III–IV. The original hypothesis was that distance to the fingerprint would differ between multiple groups with less favourable outcomes having larger distance. However, similar to our previous work,^26^ we were only able to detect increased distance in the patients with Engel III–IV outcome. While this is clinically significant, we acknowledge that the ability to distinguish between patients with Engel Ia and the other groups would have provided important additional clinical information.

There are several other important points to consider in this work. First, the sample size is small and the data come from a single centre. Specifically, the fingerprint was created using only nine seizure-free patients, which may not be enough to capture the full scope of potential network variability that could result in seizure-free outcome. However, the surgical techniques and side of surgery were varied to purposely generalize to more patients, and the evaluation was performed on a completely independent, left-out data set. Second, the analyses ultimately were constrained to 14 *a priori* nodes and no formal parameter fitting was performed. Instead, these analyses proved the hypothesis that the similarities across a small set of patients with seizure-free outcome can inform other new patients. Future work could involve a large multicentre trial with enough patients to optimize specific parameters of the fingerprint using a formal one-class classifier method.^48^ Third, as typical for this patient population, the fraction of patients with Engel III–IV outcomes is relatively small compared to those with Engel I outcome. Fourth, this work only reports seizure outcomes 1-year post-surgery. It is well known that outcomes are dynamic, and seizure-free outcomes decrease as time increases after surgery.^49^ Further work is need to understand how these networks relate to later seizure recurrence. Fifth, the method can be applied to data acquired by most 3Tesla MRI scanners with ∼30 min of scan time but it requires automated image post-processing. It may also require healthy controls scanned at the institution. Finally, multi-site validation and optimization is necessary to move this to clinical use.

## Conclusions

TLE presents a unique situation where confident clinical localization of the seizure focus does not always result in a seizure-free or favourable outcome after surgery. In this work, we demonstrate a connectome fingerprint that represents a network pattern across the brain that is associated with favourable (Engel I–II) outcome with 100% sensitivity and 90% specificity in a population of completely independent patients. Further, we show that patients with unfavourable outcomes can differ from the fingerprint in functional, structural, magnitude, and pattern of connectivity in these networks. In addition, our results support the ability of the fingerprint to provide quantitative and clinically interpretable and significant information not captured by standard clinical assessments alone or in combinations. Overall, we conclude that this automated and simple method may improve patient specific prediction of seizure outcome in patients with TLE.

## Abbreviations

FC =: functional connectome
SC =: structural connectome
RETROICOR =: Retrospective Correction of Physiological Motion Effects in functional MRI
SEEG =: stereo-encephalography
TLE =: temporal lobe epilepsy
